# Signatures of selection in the human antibody repertoire: Selective sweeps, competing subclones, and neutral drift

**DOI:** 10.1073/pnas.1814213116

**Published:** 2019-01-08

**Authors:** Felix Horns, Christopher Vollmers, Cornelia L. Dekker, Stephen R. Quake

**Affiliations:** ^a^Biophysics Graduate Program, Stanford University, Stanford, CA 94305;; ^b^Department of Bioengineering, Stanford University, Stanford, CA 94305;; ^c^Department of Pediatrics, Stanford University, Stanford, CA 94305;; ^d^Department of Applied Physics, Chan Zuckerberg Biohub and Stanford University, Stanford, CA 94305;; ^e^Chan Zuckerberg Biohub, San Francisco, CA 94158

**Keywords:** Adaptive immunity, Somatic evolution, Population genetics

## Abstract

Antibodies are created and refined by somatic evolution in B cell populations, which endows the human immune system with the ability to recognize and eliminate diverse pathogens. However, the evolutionary processes that sculpt antibody repertoires remain poorly understood. Here, using an unbiased repertoire-scale approach, we show that the population genetic signatures of evolution are evident in human B cell lineages and reveal how antibodies evolve somatically. We measured the dynamics and genetic diversity of B cell responses in five adults longitudinally before and after influenza vaccination using high-throughput antibody repertoire sequencing. We identified vaccine-responsive B cell lineages that carry signatures of selective sweeps driven by positive selection, and discovered that they often display evidence for selective sweeps favoring multiple subclones. We also found persistent B cell lineages that exhibit stable population dynamics and carry signatures of neutral drift. By exploiting the relationship between B cell fitness and antibody binding affinity, we demonstrate the potential for using phylogenetic approaches to identify antibodies with high binding affinity. This quantitative characterization reveals that antibody repertoires are shaped by an unexpectedly broad spectrum of evolutionary processes and shows how signatures of evolutionary history can be harnessed for antibody discovery and engineering.

## Significance

The immune system represents a compelling example of evolution in action: antibody diversity is created by a variety of molecular mechanisms, and then selection acts to preserve and propagate the most useful antibodies. We have combined immune repertoire sequencing with population genetics to measure the strength of selection on various antibody lineages in humans who have been vaccinated for influenza.

Antibodies are created through evolutionary processes involving mutation and selection, all of which unfold in B cell populations. As proposed by Burnet in his “clonal selection theory” in 1957, the concepts of population genetics offer an avenue for understanding how antibody repertoires evolve (1). However, after 60 years of progress in immunology, the somatic evolution of human antibodies remains poorly understood and immunology has yet to benefit from the quantitative theories and models of population genetics which have been transformative in our understanding of evolution at the organism level.

During affinity maturation, selective processes focus the antibody repertoire on antibodies that bind antigens with high affinity (2–4). After infection or immunization, activated B cells migrate to germinal centers (GCs), where they undergo genetic diversification via somatic hypermutation and selection for affinity-enhancing mutations. Within several weeks after antigenic challenge, this Darwinian process generates antibodies with increased average affinity to the antigen (5, 6). Despite intense experimental effort focused on the cellular and molecular mechanisms of affinity maturation (7–12), the evolutionary process itself remains poorly characterized. Each GC is founded by tens to hundreds of distinct B cell clones, and this diversity is often lost due to competition between clones as affinity maturation proceeds (13). However, how competition unfolds between genetically diverse variants within the same clonal B cell lineage has not been described, despite its importance for the emergence of protective antibodies. Furthermore, while signatures of selection are manifest in patterns of nucleotide substitutions when measured as bulk averages across many clonal B cell lineages (14–16), this level of resolution does not allow examination of the evolutionary histories of single clonal lineages and individual sequences within those lineages, which may have utility for antibody discovery. Although it is often presumed that the same evolutionary processes affect B cells across the entire repertoire, some B cell types, such as B-1 cells, do not participate in classical affinity maturation, and little is known about the diversity of evolutionary processes that shape distinct clones within antibody repertoires.

Here, we characterize the dynamics and somatic evolution of human B cell lineages using high-throughput sequencing of the antibody repertoire and analytical methods inspired by population genetics. We performed time-resolved measurements of antibody repertoires in healthy young adults before and after seasonal influenza vaccination. We identified vaccine-responsive B cell lineages that expanded dramatically after vaccination, and we show that patterns of genetic variation within these lineages reflect a history of strong positive selection (*SI Appendix*, Fig. S5). This selection drove recurrent selective sweeps during somatic evolution, in which antibody variants repeatedly arose via mutation and selectively expanded to become dominant within the clonal population. Many vaccine-responsive B cell lineages display evidence for selective sweeps favoring multiple subclones. Other abundant B cell lineages exhibit stable population dynamics and lack a response to vaccination; we show that these lineages carry signatures of neutral evolution (*SI Appendix*, Fig. S5). Finally, we present an approach for using phylogenetic information to identify potential high-affinity antibodies and affinity-enhancing mutations. Our results offer a detailed portrait of the somatic evolutionary processes that shape human antibody repertoires and link models of evolution with quantitative measurements of the human immune system.

**Fig. 1.**
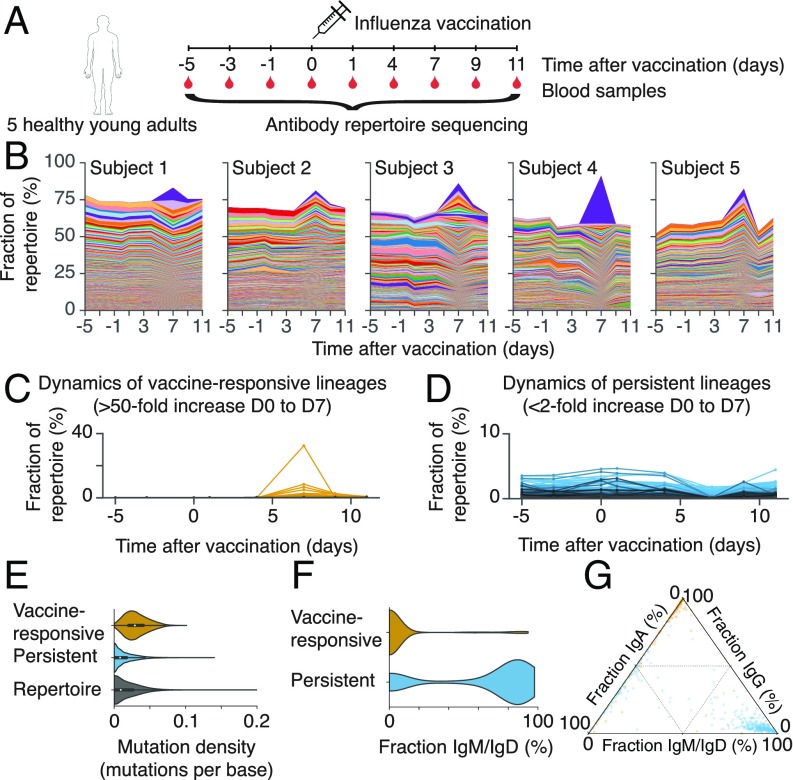
Dynamics and molecular features of antibody repertoires. (*A*) Schematic of experiment design. (*B*) Dynamics of antibody repertoires. Each line represents a clonal B cell lineage, and its width indicates the fractional abundance of that lineage (the number of unique sequences belonging to the lineage divided by the number of unique sequences in the entire repertoire) at a given time. Colors indicate distinct lineages (colors are reused across panels corresponding to different subjects and do not indicate shared sequences across subjects). The most abundant 500 lineages within each subject’s repertoire at D7 are shown. (*C*) Dynamics of vaccine-responsive lineages. (*D*) Dynamics of persistent lineages. (*C* and *D*) Each line represents a clonal lineage. (*E*) Distributions of somatic mutation density within the *V* gene in sequences belonging to vaccine-responsive lineages, persistent lineages, or the entire antibody repertoire. Mutations were called by comparison with the germline sequence. (*F*) Distributions of the fraction of sequences within each clonal lineage that were the IgM or IgD isotypes among vaccine-responsive and persistent lineages. (*G*) Fractions of sequences in each clonal lineage that were IgM or IgD, IgG, or IgA. Each dot is a lineage and is positioned according to the isotype composition of that lineage and colored according to identification as vaccine-reponsive (yellow) or persistent (blue).

We measured the dynamics of the antibody repertoires in five healthy young adults before and after vaccination in late spring of 2012 with the 2011–2012 trivalent seasonal flu vaccine (Fig. 1*A*). Volunteers were influenza vaccine–naïve for the 2010–2011 and 2011–2012 influenza seasons. We sampled peripheral blood at the time of vaccination and 1, 4, 7, 9, and 11 days afterward (D0, D1, D4, D7, D9, and D11), as well as 3 and 5 days before vaccination (D3 and D5). We sequenced transcripts of the immunoglobulin heavy chain gene (*IGH*) using RNA extracted from peripheral blood mononuclear cells (*SI Appendix*, *Materials and Methods*). The sequences spanned ∼100 bp of the variable region, including complementarity-determining region 3 (CDR3), enabling tracking of the dynamics of clonal B cell lineages. We used unique molecular barcoding to mitigate errors arising during library preparation and sequencing, enabling accurate measurement of genetic diversity (17).

To identify sequences that belong to the same clonal lineage, defined as those that share a common naïve B cell ancestor, we first grouped sequences having the same *V* and *J* germline genes and CDR3 length. Within each group, we identified clonal lineages by performing single-linkage clustering on the CDR3 sequence using a cutoff of 90% sequence identity—an approach that accurately partitions sequences into clones (18, 19).

To visualize how the composition of the antibody repertoire changed after vaccination, we examined the fractional abundance of clonal B cell lineages over time (Fig. 1*B*). We defined the fractional abundance of a clonal lineage as the number of unique sequences belonging to the lineage divided by the total number of unique sequences observed in the repertoire at that time point. All five subjects had a strong response to vaccination, exhibiting dramatic changes in the fractional abundance of B cell lineages within 7 days, which is characteristic of a memory recall response to vaccination (17). In each subject’s repertoire, we identified 36 ± 12 (mean ± SD, range 16–49) B cell lineages that expanded >50-fold between D0 and D7 after vaccination (Fig. 1*C* and *SI Appendix*, Table S1). In contrast, across a similar time span in the absence of vaccination (between D0 and D5), only 6 ± 4 lineages within each subject expanded to this extent (*SI Appendix*, Fig. S1*A*), which may be attributable to exposure to environmental antigens. Because most of these “vaccine-responsive” lineages were undetectable before vaccination, the identification of vaccine-responsive lineages was robust to the specific choice of fold-change (FC) cutoff (*SI Appendix*, Fig. S1*A*). Together, these vaccine-responsive lineages accounted for 22 ± 12% (mean ± SD, range 10–43%) of each subject’s repertoire during peak response at D7. Vaccine-responsive antibodies have high levels of somatic mutation (Fig. 1*E*) and are predominantly class-switched (Fig. 1 *F* and *G*), as expected for memory B cells. The use of germline *V* and *J* gene segments in vaccine-responsive lineages is similar to the use of the entire repertoire, with only *IGHV1-2* being significantly overrepresented among vaccine-responsive lineages (3.3-fold enrichment; *P* = 0.002, Fisher’s exact test, two-sided; *SI Appendix*, Fig. S1 *D* and *E*). We concluded that influenza vaccination triggers rapid recall of dozens of clonal B cell lineages in healthy human adults.

We discovered that each subject harbored a distinct set of clonal B cell lineages that exhibited high abundance throughout the study and were unresponsive to vaccination (<2-fold increase from D0 to D7 and >0.1% fractional abundance at D7; Fig. 1*D*). In each subject, we detected 83 ± 23 (mean ± SD, range 44–111) of these “persistent lineages,” which together accounted for 22 ± 8% (mean ± SD, range 10–33%) of the repertoire at any time point (*SI Appendix*, Table S1). Persistent lineages displayed remarkably stable population dynamics compared with vaccine-responsive lineages (*SI Appendix*, Fig. S1*B*), implying balance in cellular turnover and mRNA expression levels. Persistent antibodies have low levels of somatic mutation (Fig. 1*E*) and are mostly the IgM isotype, but a minority of persistent lineages are composed predominantly of the IgA isotype (Fig. 1 *F* and *G*). Use of germline *V* and *J* gene segments in persistent lineages is highly skewed compared with the entire repertoire: *IGHV1-69*, *IGHV3-11*, and *IGHV3-23* are significantly overrepresented (2.4-fold, 2.8-fold, and 13.6-fold enrichment, respectively; *P* < 0.001, Fisher’s exact test, two-sided; *SI Appendix*, Fig. S1*D*). *IGHJ4* was used in the vast majority of persistent lineages (86%), unlike the lineages in the rest of the repertoire (34%; 2.6-fold enrichment; *P* < 10^−107^; *SI Appendix*, Fig. S1*E*). Thus, many human antibody repertoires possess a large complement of persistent B cell lineages, which have stable population dynamics on timescales of weeks and do not respond dynamically to influenza vaccination.

**Fig. 2.**
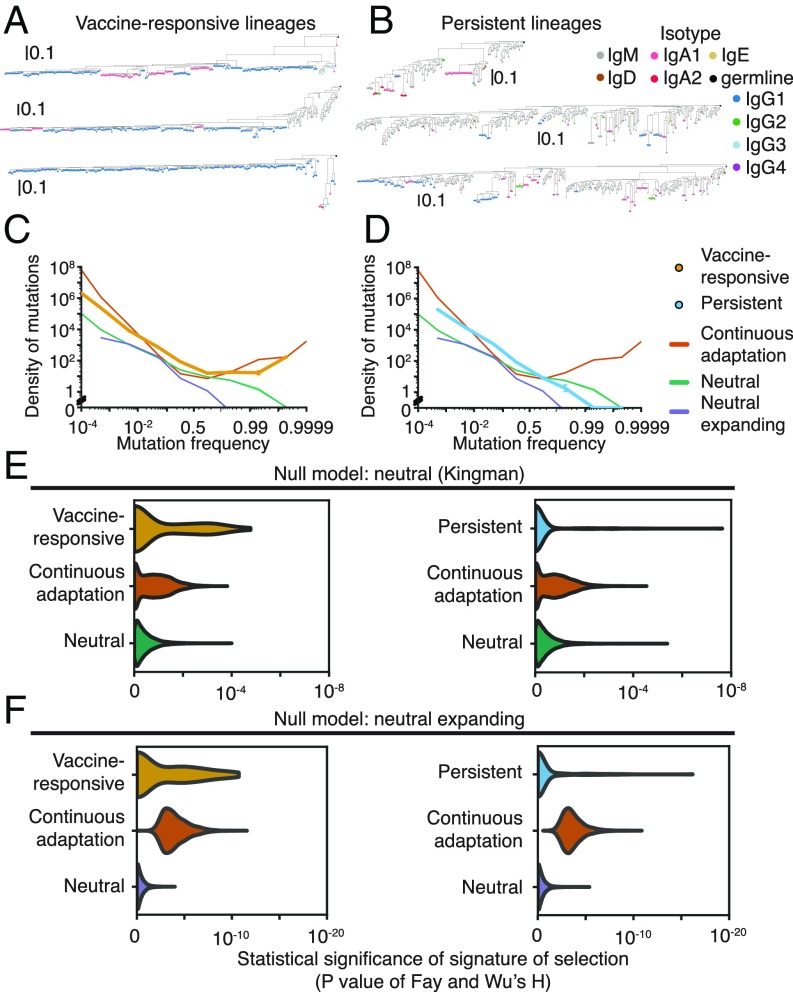
Genetic signatures of somatic evolution in clonal antibody lineages. (*A* and *B*) Examples of phylogenies of vaccine-responsive (*A*) or persistent (*B*) clonal B cell lineages. Leaves are colored by isotype. Phylogenies are rooted on the germline sequence. (*C* and *D*) SFSs averaged across all vaccine-responsive lineages (*C*) or persistent lineages (*D*). Error bars indicate SEM. SFSs generated by population genetic models of continuous adaptation driven by strong positive selection (orange), neutral drift with a constant population size (green), and neutral drift with an expanding population (purple) are shown for comparison. Shading indicates SEM across simulations (100 replicates). (*E* and *F*) Distribution of significance scores of Fay and Wu’s H statistic for vaccine-responsive lineages or persistent lineages compared against null models of neutral evolution with constant (*E*) or expanding (*F*) population size. Distributions for the null models and for simulated populations undergoing continuous adaptation driven by strong selection are also shown. Simulations were performed using population sizes sampled from the observed population-size distributions of vaccine-responsive or persistent lineages (10,000 replicates).

Evolutionary history leaves enduring signatures in the genetic diversity of populations. Vaccine-responsive B cell lineages carrying memory B cells underwent affinity maturation when the subjects were exposed to influenza antigens for the first time. We reasoned that examination of the patterns of genetic variation within these lineages might give insight into the evolutionary processes that unfolded during affinity maturation. Visualizing the phylogenies of clonal B cell lineages revealed that many vaccine-responsive lineages possess a highly imbalanced branching structure across many levels of depth, suggesting that these lineages experienced recurrent selective sweeps (Fig. 2*A*). This signature, reflecting continuous adaptive evolution under strong positive selection, has been found in many asexual populations evolving under sustained adaptive pressure, such as influenza virus (20) and HIV (21).

To quantitatively characterize the evolutionary histories of clonal B cell lineages, we examined the frequency spectrum of derived somatic mutations, also known as the site frequency spectrum (SFS). The SFS carries detailed information about evolutionary history and can be useful for detecting selective processes from snapshot sampling of genetic diversity (*SI Appendix*, Fig. S5). In continuously adapting asexual populations, the SFS exhibits a distinct excess of high-frequency variants, which can be used to rule out neutral models and infer positive selection (22), as in the cases of influenza virus (20) and HIV (21). We calculated the SFS of each clonal B cell lineage based on somatic point mutations relative to the personalized germline *V* and *J* gene sequences for each subject because the ancestral state is known with high confidence for these sites (*Materials and Methods* and *SI Appendix*, Fig. S1*C*). We compared the observed SFSs against population genetic models of neutral evolution with constant population size [Kingman coalescent (23)], neutral evolution with expanding population size, and continuous adaptation [Bolthausen-Sznitman coalescent (24)] using computer simulations (*SI Appendix*, *Materials and Methods*).

We first visualized the SFS as an average over all vaccine-responsive lineages and found that the SFS was highly skewed, exhibiting a large excess of high-frequency somatic mutations in clear disagreement with the neutral model (Fig. 2*C*). Instead, the model of positive selection had an excellent fit to the data, implying that the dominant mode of evolution in vaccine-responsive lineages is continuous adaptation occurring via recurrent selective sweeps driven by the occurrence of beneficial mutations. Furthermore, this pattern cannot be explained by neutral expansion of a population, which was previously shown (25) and which we confirmed using simulations (Fig. 2*C*). This finding is consistent with the classical model of affinity maturation: Affinity-enhancing mutations arise, and selection focuses the repertoire on these variants, driving the loss of intraclonal diversity. The presence of deep branches harboring persistent minor alleles within each clonal lineage indicates that memory B cells frequently exit GCs while selection continues, preventing complete loss of diversity due to selective sweeps. These signatures likely reflect historical positive selection during the primary immune response, rather than the recall response, because formation of GCs during the memory response occurs at longer timescales of several weeks (26).

Next, we sought to characterize the patterns of somatic evolution at the resolution of individual clonal B cell lineages. While individual lineages have fewer somatic mutations and thus exhibit sparse spectra compared with population averages, we found that many vaccine-responsive lineages have a large excess of high-frequency mutations (*SI Appendix*, Fig. S2*A*). To quantitatively detect selection, we used Fay and Wu’s (27) H statistic, which was originally devised to detect high-frequency hitchhiking alleles that are transiently associated with selective sweeps in recombining populations, but can also sensitively detect selective sweeps in asexual populations. Using H, we found that 32% of vaccine-responsive lineages deviate significantly from the neutral model with constant population size (Fig. 2*E* and *SI Appendix*, Fig. S2 *B* and *C*; *P* < 0.05). Similarly, 43% of vaccine-responsive lineages deviate significantly from the neutral model with population expansion (Fig. 2*F*; *P* < 0.05). We also directly measured the nonmonotonicity of the SFS and found that 14% of vaccine-responsive lineages deviated significantly from neutrality by this alternative metric for selection (*SI Appendix*, Fig. S2 *D* and *E*). Nearly every subject had at least one vaccine-responsive lineage that evidently experienced selection (*SI Appendix*, Fig. S2*G*).

The failure to detect selection in every vaccine-responsive lineage is consistent with statistical limits of detection arising from the population sizes of the lineages (*SI Appendix*, Fig. S2*F*). Indeed, selection was detected at a rate that is consistent with a model in which every vaccine-responsive lineage evolved under strong positive selection (*SI Appendix*, Fig. S4*A*), suggesting that the sensitivity of the statistical test, given the sizes of the sampled populations, accounted for the failure to detect selection in every vaccine-responsive lineage. In support of this, the signature of selection, as measured by the significance of Fay and Wu’s H compared with size-matched neutrally evolving lineages, showed a trend toward inverse correlation with the number of sampled sequences in the lineage (*SI Appendix*, Fig. S4*C*; Spearman’s rho = −0.08, *P* = 0.09). In turn, the number of sequences in the lineage correlated strongly with the total amount of nucleotide diversity (*SI Appendix*, Fig. S4*D*; Pearson’s R = −0.51, *P* < 10^−19^), suggesting that reliable detection of selection relies on having sufficient mutational diversity to support phylogenetic analysis.

High-frequency derived mutations are enriched within complementarity-determining regions (CDRs), which form the antibody-antigen binding interface and often evolve under positive selection (14, 15). Such mutations are depleted in framework regions (FWRs; *SI Appendix*, Fig. S2*I*), which form the structural scaffold of the antibody molecule and typically evolve under purifying selection (14, 15). Together these observations demonstrate that evolutionary history can be quantitatively characterized at the resolution of individual clonal B cell lineages; also, they support the conclusion that vaccine-responsive lineages evolved under continuous adaptive pressure on antibody-antigen interactions.

Persistent antibody lineages have a strikingly different mode of evolution. When we visualized the SFS as an average over all persistent lineages, we found its shape to be consistent with neutral evolution, lacking an excess of high-frequency somatic mutations (Fig. 2*D*). Indeed, persistent lineages had no mutations at frequencies above 99%, in agreement with the prediction of the neutral model but not the model of positive selection. This pattern was also clearly evident in individual clonal lineages (*SI Appendix*, Fig. S3*A*) as reflected in their balanced phylogenies, which are consistent with the absence of selection and characteristic of neutral drift-like evolution (Fig. 2*B*). Using Fay and Wu’s H statistic, we found that nearly every persistent lineage (94%) had no significant departure from the neutral model with constant population size (Fig. 2*E* and *SI Appendix*, Fig. S3 *B* and *C*; *P* > 0.05). Similarly, 88% of persistent lineages had no significant deviation from the neutral model with population expansion (Fig. 2*F*; *P* > 0.05). We also found no significant departure from neutrality for nearly every persistent lineage (99%) using the nonmonotonicity of the SFS as a metric for selection (*SI Appendix*, Fig. S3 *D* and *E*). Persistent lineages had large population sizes comparable to those of vaccine-responsive lineages (100 to ∼11,000 sequences; *SI Appendix*, Fig. S3*F*), indicating that limits of detection arising from population size cannot explain the failure to detect selection. Indeed, the rate at which we detected selection on persistent lineages was much lower than the detection limit (*SI Appendix*, Fig. S4*B*). Thus, persistent lineages evolve in a manner consistent with neutrality, suggesting that neutral birth-death processes are responsible for the expansion and maintenance of a substantial fraction of the human antibody repertoire.

The molecular features of persistent lineages are characteristic of B-1 cells, a B cell subtype that has a different life history than the better-studied B-2 cells. Both persistent lineages and B-1 cells are mostly IgM (28), with a minority of lineages composed predominantly of IgA (29) (Fig. 1 *F* and *G*), and have low levels of somatic hypermutation (Fig. 1*E*), consistent with a life history lacking a stage of classical affinity maturation. B-1 cells are thought to constitute a separate B cell population having distinct progenitors (30), consistent with our observation that the persistent lineages form a distinct set of clonal lineages. If persistent lineages are indeed derived from B-1 cells, our results suggest that expansion and maintenance of B-1 cell populations are neutral processes, in sharp contrast to the strong positive selection that shapes vaccine-responsive B cells. The molecular identity of human B-1 cells has been elusive (31, 32), and our prediction that these cells are distinguished by the genetic signatures of somatic evolution opens a new avenue for identification and characterization of this cell population.

**Fig. 3.**
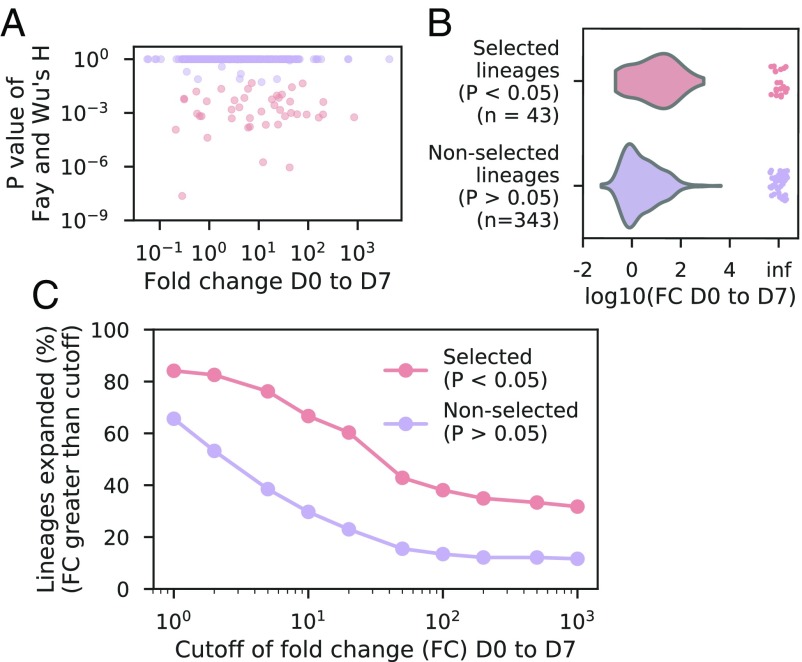
Relationship between genetic signatures of selection and clonal expansion after vaccination. (*A*) Signatures of selection compared with magnitude of clonal expansion after vaccination. Each dot is a lineage. Color indicates whether a lineage has a significant signature of selection, as indicated by the legend in *C*. (*B*) Distribution of magnitudes of clonal expansion after vaccination [fold-change (FC) from D0 to D7] among selected and nonselected lineages. Points at “inf” indicate lineages that were detected at D7 but not at D0 and therefore have undefined FC. (*C*) Fraction of lineages that exhibited clonal expansion with magnitude exceeding various cutoffs among selected and nonselected lineages.

Next, we studied how the genetic signatures of selection relate to clonal expansion after vaccination. In this analysis, we considered all clonal families having at least 100 sequences at D7 regardless of their extent of clonal expansion after vaccination. This included all vaccine-responsive and persistent lineages, as well as other lineages that expanded less than the FC cutoff for vaccine-responsive lineages (50-fold) but more than the FC cutoff for persistent lineages (2-fold), yielding a total of 450 lineages. We found that positive selection is highly correlated with clonal expansion (Fig. 3*A*; Spearman’s rho = −0.27, *P* < 10^−9^). Lineages with significant evidence of positive selection (*P* < 0.05 in comparison with a neutral model with constant population size) expand more after vaccination than lineages without such evidence (Fig. 3*B*; median FC from D0 to D7 of selected lineages = 1.05, nonselected lineages = 0.23; *P* < 10^−4^, Mann-Whitney *U* test, two-sided). Furthermore, regardless of the choice of FC cutoff for defining clonal expansion, many more positively selected lineages than nonpositively selected lineages undergo clonal expansion (Fig. 3*C*). These results indicate that memory recall after vaccination predominantly involves clonal expansion of positively selected lineages. However, we note that not all positively selected lineages undergo clonal expansion, as expected given the presence of affinity-matured memory B cell lineages having specificity for other antigens besides influenza. Conversely, some lineages that evidently evolved neutrally also undergo clonal expansion after vaccination, suggesting that memory B cell activation and expansion are not necessarily linked to a history of affinity maturation.

How is the clonal structure of individual B cell lineages influenced by selection? During affinity maturation, subclones harboring independent mutations within a B cell lineage compete for evolutionary success. Competition can result in either one winner or multiple winners within a clonal lineage. Multiple winners may arise due to independent competition in spatially separated regions, such as different GCs, or because subclones harboring different beneficial mutations compete to a stalemate within the same GC, a scenario known as “clonal interference” (33). To further dissect the evolutionary processes of affinity maturation, we characterized the clonal structures of vaccine-responsive lineages.

**Fig. 4.**
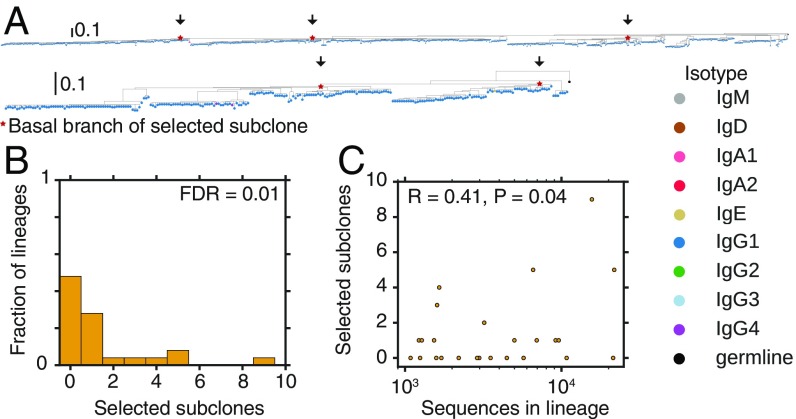
Signatures of selective sweeps within multiple subclones of vaccine-responsive antibody lineages. (*A*) Examples of phylogenies of vaccine-responsive clonal B cell lineages having evidence for selective sweeps favoring multiple subclones. Clades identified as significantly positively selected by our algorithm (*P* < 0.05) are indicated by arrows and red stars. Leaves are colored by isotype. Phylogenies are rooted on the germline sequence. (*B*) Distribution of the number of distinct selected subclones (i.e., clades displaying evidence for a selective sweep) within vaccine-responsive lineages having >1,000 sequences. FDR, false discovery rate. (*C*) Relationship between the number of distinct selected subclones within a clonal lineage and population size (number of sequences) of the lineage. Pearson correlation coefficient is shown.

Using phylogenetic analysis, we found that many vaccine-responsive clonal B cell lineages contain multiple positively selected subclones. While some phylogenies harbor only one imbalanced clade displaying characteristics of recurrent selective sweeps (Fig. 2*A*), others have several large clades that each exhibit these characteristics, suggesting that multiple subclones persisted as winners within these clonal lineages (Fig. 4*A*). To quantify this phenomenon, we developed an algorithm to identify and count positively selected subclones in an unbiased manner (*SI Appendix*, *Materials and Methods*). We found that 24% of vaccine-responsive lineages composed of >1,000 sequences harbor multiple subclones that have evidence of positive selection (Fig. 4*B*; false discovery rate of 1%). This indicates that affinity maturation often focuses the repertoire onto multiple subclones arising from a common B cell ancestor. These subclones share somatic mutations that were acquired before branching in every case, which is evidence against these results being artifacts arising from erroneous joining of nonclonal sequences during lineage reconstruction. The number of selective sweeps within a lineage is modestly but significantly correlated with the population size of the lineage (Fig. 4*C*), suggesting that clonal amplification of very large B cell lineages often involves selection favoring multiple subclones. Previous reports indicate that clonally related sequences are occasionally found in distinct GCs located within the same lymph node (13), suggesting a role for spatial segregation in facilitating independent selection of subclones.

Because B cell fitness is tightly coupled to antibody affinity during affinity maturation, we hypothesized that the genetic diversity of B cell populations encodes information about binding affinity. Amplification of highly fit variants can be readily observed in phylogenies, and elevated fitness is thought to be associated with enhanced antibody affinity. Therefore, we sought to leverage phylogenetic signals that reveal the fitness of individual antibody sequences to identify candidate high-affinity antibodies and affinity-enhancing mutations based on sequencing data alone. Specifically, we used a computational approach to infer the fitness of sequences based on their phylogenetic context (34) and then identified sequences that had high fitness.

**Fig. 5.**
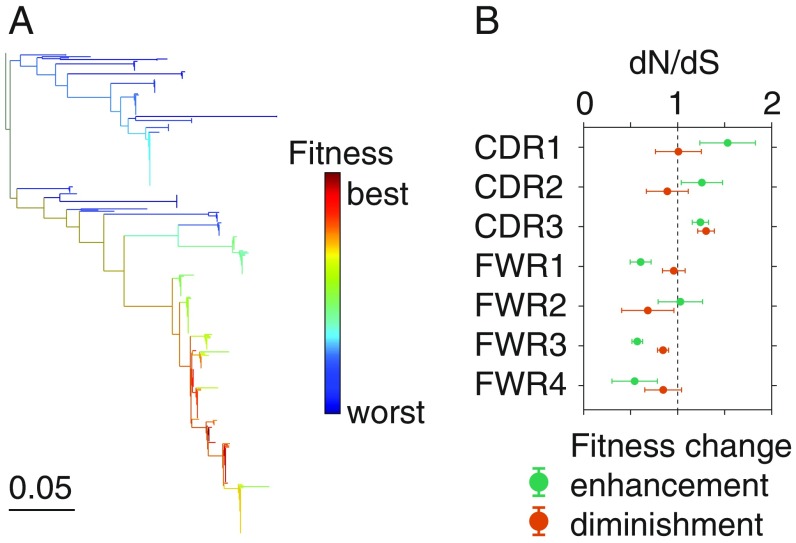
Phylogenetic identification of affinity-enhancing mutations. (*A*) Example of a phylogeny of a clonal B cell lineage colored by the inferred fitness of each sequence. (*B*) Regional distribution of nonsynonymous mutations associated with strong fitness enhancements (top three branches ranked by fitness change from parent to child) or diminishments (bottom three branches ranked by fitness change from parent to child), displayed as enrichment relative to synonymous mutations (dN/dS) in the same branches. Dashed line indicates no enrichment. Error bars indicate one SD as determined by bootstrap (100 replicates).

In line with a history of selective sweeps, phylogenetic inference revealed wide variation in fitness among sequences within vaccine-responsive B cell lineages, with some sequences predicted to have much higher fitness than other sequences in the same clonal lineage (Fig. 5*A*). We identified mutations associated with the strongest fitness enhancements (top three branches ranked by fitness change from parent to child sequence in each lineage) (*SI Appendix*, *Materials and Methods*). In comparison with synonymous mutations, nonsynonymous fitness-enhancing mutations were highly enriched in CDRs (Fig. 5*B*; *P* < 0.008 for CDR1, *P* < 0.1 for CDR2, and *P* < 2 × 10^−6^ for CDR3; Fisher’s exact test, two-sided) and depleted in FWRs (*P* < 0.009 for FWR1, *P* < 2 × 10^−11^ for FWR3, and *P* < 0.01 for FWR4) with the sole exception of FWR2 (*P* = 0.87). Thus, phylogenetic inference of fitness enhancement-associated mutations is consistent with the expected distribution of nonsynonymous and synonymous mutations in the tree based on the structural basis of antibody-antigen interactions (35–37). This finding supports the functional relevance of the identified fitness enhancement-associated nonsynonymous mutations. Mutations associated with the strongest fitness diminishments (bottom three branches in each lineage) were also enriched in CDR3 (Fig. 5*B*; *P* < 8 × 10^−11^), consistent with the idea that mutations in CDRs, especially CDR3, can sometimes harm fitness because they disrupt antibody-antigen binding interfaces, suggesting that the traditional notion of purifying selection being confined to FWRs is overly simplistic. While these predictions must be validated experimentally via expression of antibodies with native heavy and light chain pairing, our results suggest that phylogenetic methods can reveal information about antibody affinity which is encoded in sequence diversity and potentially can be used to rapidly identify high-affinity antibodies and affinity-enhancing mutations.

In summary, our results demonstrate that human antibody repertoires are shaped by a broad spectrum of somatic evolutionary processes. Prior efforts to detect selection in antibody genes focused on regions or residues in aggregate across many clonal B cell lineages (14–16), and did not account for the fact that evolution acts differently on different clonal lineages. On the other hand, prior studies of the antibody repertoire response after vaccination did not focus on the molecular signatures of selection (17, 18, 38, 39). We characterized signatures of selection within individual clonal B cell lineages up to the fundamental limits imposed by their population size, revealing that a diversity of evolutionary modes exists within the B cell repertoire. Vaccine-responsive lineages display pervasive evidence of positive selection, and many lineages experience selective sweeps favoring multiple subclones, suggesting that subclonal competition is common during affinity maturation. While our results support competition within clonal lineages, it is likely that competition between clonal lineages also exists. These signatures likely reflect selection during affinity maturation, which is often directed toward viral antigens seen during early life (40, 41).

On the other hand, persistent lineages display signatures of neutral drift-like evolution, revealing that nonselective processes generate a substantial fraction of human antibody repertoires and requiring that the conventional notion that selective processes are ubiquitous in antibody maturation be modified. This diversity of evolutionary modes likely reflects the diversity of life histories among distinct B cell types. The presence of large clonal lineages lacking molecular signatures of selection also provides an inherent control and constitutes evidence that the detection of such signatures in vaccine-responsive lineages is not an artifact of our approach, including a failure to correctly determine the germline sequence. In addition, the presence of large clonally expanded persistent lineages evidently displaying signatures of neutral drift indicates that population expansion itself cannot account for the signatures of selection observed in vaccine-responsive lineages. Importantly, our results suggest that the molecular signatures of selection distinguish vaccine-responsive lineages from other clonal lineages that are also highly abundant after vaccination. We have shown that molecular signatures of selection can be harnessed through phylogenetic approaches to identify sequences that were most favored by selection during affinity maturation and therefore likely encode high-affinity antibodies with potential utility for biomedical applications. High-throughput sequencing of human antibody repertoires and analysis through the lens of population genetics thus offer a promising avenue for antibody discovery and engineering.

## Materials and Methods

All study participants gave informed consent, and protocols were approved by the Stanford Institutional Review Board. Five healthy humans aged 18–28 were vaccinated with the 2011–2012 seasonal trivalent inactivated influenza vaccine and gave blood 3 and 5 days before vaccination (D3 and D5), immediately before vaccination (D0), and 1, 4, 7, 9, and 11 days afterward (D1, D4, D7, D9, D11). Peripheral blood mononuclear cells were isolated, total RNA was extracted, and sequencing libraries were prepared from 500 ng of total RNA using isotype-specific *IGH* constant region primers for reverse transcription and *IGH* variable region primers for second-strand cDNA synthesis followed by PCR, following Vollmers et al. (17) and Horns et al. (18). Sequencing was performed for all libraries using the Illumina HiSeq 2500 or MiSeq platform with paired-end reads. Sequences were preprocessed using a custom informatics pipeline to perform consensus unique molecular identifier (UMI)-based error correction, annotation of *V* and *J* gene use and CDR3 length using IgBLAST (42), and isotype determination using BLASTN. Clonal lineages were identified by grouping sequences sharing the same *V* and *J* germline genes and CDR3 length, and then performing single-linkage clustering with a cutoff of 90% nucleotide identity across both the CDR3 and the rest of the variable region (18). SFSs were constructed based on somatic mutations relative to the germline *V* and *J* genes (excluding CDR3 polymorphisms because the ancestral state may not be known with high confidence in the CDR3) and then compared with simulations of evolutionary models using betatree (43) or custom software. Multiple sequence alignment was performed using a custom fast heuristic algorithm based on MUSCLE (44), and phylogenetic reconstruction was performed using FastTree (45). Selection on subclones was detected using a custom algorithm that performs greedy breadth-first search based on Fay and Wu’s H statistic (27) of subtrees. Fitness inference based on the local branching rate of a phylogeny was performed following Neher et al. (34).

## Supplementary Material

Supplementary File
