# Supplementary material for: Signatures of selection in the human antibody repertoire: Selective sweeps, competing subclones, and neutral drift
Source: Proc Natl Acad Sci U S A. 2019 Jan 8;116(4):1261–6. doi: 10.1073/pnas.1814213116 (PMC6347681; doi:10.1073/pnas.1814213116)
Supplement: Supplementary File [file pnas.1814213116.sapp.pdf]

PNAS
www.pnas.org

Supplementary Information for

Signatures of Selection in the Human Antibody Repertoire: Selective Sweeps, Competing Subclones, and Neutral Drift

Felix Horns, Christopher Vollmers, Cornelia L. Dekker, Stephen R. Quake

Corresponding author: Stephen R. Quake
Email: quake@stanford.edu

This PDF file includes:

Materials and Methods
Figs. S1 to S5
Tables S1 to S2
References for SI reference citations

www.pnas.org/cgi/doi/10.1073/pnas.1814213115

Materials and Methods

Study participants
All study participants gave informed consent and protocols were approved by the Stanford Institutional Review Board. Five humans aged 18-28, including 3 males and 2 females, were recruited in 2011. All subjects were apparently healthy and showed no signs of disease.

Sample collection
Blood was drawn by venipuncture. Peripheral blood mononuclear cells (PBMCs) were isolated using a Ficoll gradient and frozen in 10% (vol/vol) DMSO and 40% fetal bovine serum (FBS) according to Stanford Human Immune Monitoring Center protocol. Subjects were vaccinated with the 2011–2012 seasonal trivalent inactivated influenza vaccine. Blood was collected 3 and 5 days before vaccination (D-3 and D-5); immediately before vaccination (D0); and 1, 4, 7, 9, and 11 days afterwards (D1, D4, D7, D9, D11).

RNA extraction and library preparation
PBMCs were thawed on ice and total RNA was extracted using the Qiagen AllPrep kit (Valencia, CA) following manufacturer's instructions. Sequencing libraries were prepared from samples at all time points using 500 ng of total RNA as input following the protocol described in (1). Briefly, RNA was annealed to a pool of six isotype-specific IGH constant region primers containing 8 random nucleotides (nt), which serve as a molecular barcode for consensus error correction, by incubation at 72 C for 3 min, then placed on ice for 2 min. First-strand cDNA synthesis was performed using Superscript III reverse transcriptase (Life Technologies, Carlsbad, CA) following manufacturer's protocol. Second-strand cDNA synthesis was performed using Phusion HiFi DNA polymerase (Thermo Scientific, Waltham, MA) and a pool of ten IGH variable region-specific primers containing 8 random nt (98°C for 4 min, 52°C for 1 min, 72°C for 5 min). Double-stranded cDNA product was purified twice using Ampure XP beads (1:1 ratio) (Beckman Coulter, Indianapolis, IN). Amplification was performed using Platinum Hifi DNA polymerase (Life Technologies, Carlsbad, CA) and primers containing Illumina sequencing adapters and dual sample indexes. Products were purified using Ampure XP beads (1:1 ratio), then pooled for multiplexed sequencing.

We prepared additional sequencing libraries from D7 samples following the protocol described in (2). This protocol is identical to that described above, except that ten isotype subtype-specific IGH constant region primers and six IGH variable region-specific primers each containing 8 or 12 random nt were used. Products are longer amplicons spanning most of the IGH variable region and ~100 bp of the IGH constant region. We used a different aliquot of total RNA from the same D7 samples as input. All PCR primer sequences are provided in Table S2.

Sequencing
Sequencing was performed for libraries from all time points using the Illumina HiSeq 2500 platform (San Diego, CA) using paired-end 101 bp reads. For libraries prepared from the D7 time point with longer amplicons, sequencing was performed using the Illumina Miseq platform using paired-end 300 bp reads. We obtained 826,472 ± 413,841 reads (mean ± s.d., range 170,477 – 1,988,165) for each library.

Preprocessing of sequence data
To process sequencing reads, we used a custom informatics pipeline similar to (2). Briefly, consensus sequences were constructed from reads containing the same 16 nt random barcode. Quality scores were propagated to the consensus sequence. To implement stringent filtering of sequence errors, only consensus sequences formed from >1 read were kept for further analysis. Sequences were annotated with *V* and *J* germline gene usage and CDR3 length using IgBlast (3).

Isotypes were determined using BLASTN against a custom database of IGH constant region sequences. Further error filtering was performed by removing sequences supported by only one random barcode and separated by exactly one substitution from another sequence having >500 unique molecular barcodes; these sequences likely originate from errors occurring during library preparation.

Identification of clonal B cell lineages

Sequences belonging to the same clonal B cell lineage were identified using clustering following (2). Briefly, sequences sharing the same *V* and *J* germline genes and CDR3 length were grouped. Within each group, single-linkage clustering was performed with a cutoff of 90% nt sequence identity across both the CDR3 and the rest of the variable region. Sequence identity was computed by counting mismatches in gapless pairwise sequence alignments. Quality filtering was implemented by assuming mismatches at positions where either aligned base had $Q \leq 5$. The cutoff of 90% was chosen because it is a distinct minimum in the distribution of pairwise nucleotide distances between sequences. This approach has been shown to partition sequences into clonal lineages with high sensitivity and specificity (2, 4).

Tracking dynamics of clonal B cell lineages

To track the dynamics of clonal B cell lineages, we calculated the fractional abundance of each lineage, defined as the number of unique sequences within the lineage divided by the total number of unique sequences observed in the repertoire at that time point. For this calculation, we only used reads that were sequenced using the short amplicon protocol. Vaccine-responsive lineages were identified based on the fold-change (FC) of their fractional abundance between D0 and D7 (>50-fold increase). Persistent lineages were identified as those having stable fractional abundance between D0 and D7 (<2-fold increase). We further required that each vaccine-responsive and persistent lineage represent >0.1% of the repertoire at D7 (corresponding to ~40 distinct sequences) to remove very small clonal lineages from consideration. Isotype composition and mutation density were calculated using sequences from all time points.

Identification of non-reference germline variants

To annotate non-reference germline variants in a personalized manner for each subject, we adapted the method developed by Gadala-Maria and colleagues (5). We first grouped sequences having the same *V* or *J* germline sequence. For each mutation detected by comparison against the reference germline sequence, we performed regression on the mutation frequency against the mutation count of the entire *V* or *J* segment. Specifically, we binned sequences into groups based on the number of mutations per sequence and calculated the frequency of the focal mutation in each bin. We then fit a linear model to these data using least-squares optimization. Mutations with y-intercepts greater than 0.125 at a significance level of $P < 0.05$ as assessed using Student's t test were considered potential germline variants. Because alleles might contain multiple non-reference germline variants, bins were excluded from the regression based on detection of outliers (bins having more than 1.5-times the interquartile range greater than the third quartile of the number of sequences in the bins carrying 1–10 mutations). If an outlier bin was found, then all bins having fewer mutations per sequence were excluded from the regression.

Calculation of site frequency spectrums

We constructed the site frequency spectrum (SFS) of each clonal B cell lineage based on somatic mutations relative to the germline *V* and *J* genes. For analysis of the SFS and further phylogenetic analysis, we used only reads originating from the D7 samples that were sequenced using the long amplicon protocol. Vaccine-responsive and persistent lineages having <100 unique sequences in these samples were excluded from this analysis. Mutations were called using IgBlast (3) and we removed non-reference germline variants for each individual subject as determined above. We

calculated the frequency of each mutation within a clonal lineage (number of sequences containing the mutation divided by the number of sequences in the lineage). We note that our approach conservatively excludes most mutations in the CDR3 because these mutations lie within the highly variable untemplated region of the IGH sequence and therefore the ancestral state may not be known with high confidence. Because we exclude CDR3 polymorphism, the long amplicon sequencing reads contain many more polymorphic sites, which contribute information to our phylogenetic analysis, compared to the short amplicon reads.

To visualize the SFS, we binned the mutation frequencies using bins spaced according to the logit function (inverse logistic transform). Bin edges were 10^{-5}, 10^{-4}, 10^{-3}, 10^{-2}, 10^{-1}, 0.5, 0.9, 0.99, 0.999, 0.9999, 0.99999. The mutation density within each bin was calculated by normalizing by the bin size (number of mutations in bin divided by the width of bin). To calculate the average SFS across many lineages (e.g., all vaccine-responsive lineages or vaccine-responsive lineages from one study subject), we calculated the SFS for each lineage individually, then calculated the average mutation density in each bin. Each lineage is weighted equally and therefore the average is not influenced by the population sizes or relative mutational loads of the lineages.

Use of the SFS for detecting selection has several practical advantages. Calculation of the SFS does not depend on phylogenetic reconstruction or ancestral sequence reconstruction, and the reliability of these inferences. Unlike traditional tree imbalance measures, such as the Colless or Sackin indices, the SFS is readily calculated for populations with multifurcating phylogenies, such as B cell populations. Finally, unlike approaches that require phylogenetic reconstruction, calculation of the SFS scales linearly with the number of sequences and therefore can be evaluated readily for lineages having many sequences.

We note that the behavior of the low-frequency region of the SFS cannot be directly interpreted as evidence for selection in our analysis. In our stringent error-filtering process, we removed many singleton sequences (supported by only one read or one unique molecular barcode). Because some *bona fide* singleton mutations are very likely removed, the low-frequency behavior of the SFS is affected and cannot be interpreted quantitatively as a test for selection.

Simulations of evolutionary models

To compare the observed patterns of evolution with evolutionary models, we performed simulations of beta coalescent models using the betatree package in Python (6). Specifically, we simulated neutral evolution using the Kingman coalescent ($\alpha = 2$) and evolution under strong positive selection using the Bolthausen-Sznitman coalescent ($\alpha = 1$). For comparison with the observed SFSs averaged across many lineages, we simulated ensembles of 100 lineages (similar to the number of observed vaccine-responsive lineages) each having a number of leaves sampled without replacement from the distribution of population sizes of vaccine-responsive lineages (median population size was approximately 1,000 sequences), and calculated the average SFS across these lineages (Figure 2A and Figure 2B). To model neutral evolution with population expansion, we performed forward-time simulations using custom software. Each simulation was initialized with a single individual. At each time step, one individual was chosen to reproduce by sampling uniformly at random from the population. During reproduction, mutations were introduced following a Poisson distribution with rate parameter of 0.3 (chosen based on observed per-base mutation rates due to somatic hypermutation). The simulation was terminated when the population size reached the target and the SFS was calculated based on the final mutation frequencies.

Calculation of test statistics for selection

We calculated Fay and Wu's H statistic using the counts of somatic mutations within a clonal lineage:

$$H = \hat{\theta}_\pi - \hat{\theta}_H$$

with

$$\hat{\theta}_H = \sum_{i=1}^{n-1} \frac{2S_i i^2}{n(n-1)},$$

and

$$\hat{\theta}_\pi = \sum_{i=1}^{n-1} \frac{2S_i i(n-i)}{n(n-1)}$$

,

where S_i is the number of mutations observed in *i* sequences of the lineage and *n* is the total number of sequences in the lineage, i.e. the population size of the lineage (7).

As an alternative metric for selection, we directly estimated the non-monotonicity of the high-frequency region of the SFS. Specifically, we fit a quadratic polynomial to the binned SFS using least-squares minimization, calculated its first derivative, and determined the maximum value of the first derivative in bins representing frequencies >0.25, which we define as the non-monotonicity *D*. SFSs having an excess of high-frequency mutations display a characteristic "uptick" or non-monotonicity in the high-frequency region and therefore have positive *D*.

Calculation of the statistical significance of test statistics

We evaluated the statistical significance of tests for selection by comparison with a null distribution of the test statistic generated under a neutral model of evolution (the Kingman coalescent). We simulated an ensemble of 1,000 lineages using the Kingman coalescent and calculated the test statistic (Fay and Wu's H or the non-monotonicity *D*) for each lineage. Thus, we created a distribution of the test statistic under the null model. We then fitted the Johnson's U distribution to this data. To evaluate the statistical significance of a test statistic for a focal lineage, we calculated the P value of the test statistic (that is, the probability of obtaining by chance a value of the test statistic that is at least as extreme as the given value) under the null distribution by integrating its probability density. Because population size strongly influences the distribution of test statistics, we always tested for selection by comparison against a null distribution characterizing populations of a size matched to that of the focal lineage. To accomplish this, we simulated the null distribution as described above for a range of population sizes (N = 100, 200, 500, 1000, 2000, 5000, 10000, and 20000 leaves). Given a focal lineage, we determined the nearest population size within this set and used the corresponding null distribution for comparison. We refer to this procedure as matching the population size of the focal lineage to the null distribution.

Determining the limit of detection of selection due to population size

Detection of selection is fundamentally limited by population size. The detection limit was calculated by simulating an ensemble of 1,000 lineages under strong positive selection using the Bolthausen-Sznitman coalescent model. Fay and Wu's H statistic was calculated for each lineage and its significance was assessed by comparison with the neutral model. This was repeated for populations having various sizes (N = 100, 200, 500, 1000, 2000, 5000, 10000, 20000 leaves). The fraction of lineages that were identified as significantly positively selected ($P < 0.05$) in each case is the expected rate of detecting positive selection in the scenario where all lineages are generated under strong positive selection.

Phylogenetic reconstruction
We used a fast heuristic algorithm to construct a multiple sequence alignment and reconstruct the phylogeny of each clonal lineage. Sequences were first aligned in an ungapped manner using the start and end positions of the CDR3 as anchor points. This alignment was refined using MUSCLE with "-refine -maxiters 1 -diags -gapopen -5000" (8). The large gap penalty reflects our expectation that insertions and deletions are uncommon during somatic hypermutation (9, 10). We aligned a germline sequence consisting of the concatenated *V* and *J* germline alleles of the lineage by profile-profile alignment using MUSCLE with "-profile -maxiters 1 -diags". We reconstructed the phylogeny using FastTree 2 with "-nt -gtr" (11). Finally, we performed joint refinement of the multiple sequence alignment and phylogeny by identifying extremely long branches (>0.5 substitutions/site), removing them all from the alignment, and realigning one sequence at a time by profile-profile alignment using MUSCLE with "-profile -maxiters 2 -diags", then repeating phylogenetic reconstruction as described above.

Detecting selection in multiple subclones of a clonal lineage
We developed an algorithm to identify subclones having evidence of positive selection. Our algorithm is based on calculation of the test statistic on subclones, then searching within the phylogeny to identify the largest independent subclones displaying significant evidence of selection. Specifically, we calculate Fay and Wu's H statistic on every large clade (having >100 sequences) based on the frequency of somatic mutations that occurred within the clade, and calculate its P value by comparison with the null distribution for phylogenies matched in size to the number of leaves in the clade. We then perform a greedy breadth-first search for clades having significant evidence of selection. This search strategy yields the deepest subclones having evidence of selection and guarantees that all such subclones represent mutually exclusive subsets of the lineage. We note that this is a conservative strategy because in a case where a deep clade has evidence of selection, but in turn harbors two independent subclades that themselves have evidence of selection, the search stops at the deep clade and therefore will not discover the selected subclades. To correct for multiple hypothesis testing, we adjusted the P value associated with Fay and Wu's H statistic using the Bonferroni method based on the number of tests performed during the search step.

We observed that standard tests of selection, such as Fay and Wu's H statistic, often failed to detect selection when applied to lineages harboring multiple positively selected subclones. When multiple subclones persist, the frequency of a derived mutation which is private to a single subclone has a hard upper bound, causing tests based on the presence of high frequency mutations to fail (Figure S4E). This highlights the influence of clonal population structure on tests for selection, an important design consideration for efforts to detect selection in any asexual population.

Identification of candidate affinity-increasing mutations
Using the reconstructed phylogeny of each clonal lineage as input, we performed fitness inference following (12). Fitness inference is based on the idea that nodes having higher fitness create offspring at a faster rate than other nodes and therefore the local branching rate of a phylogeny carries information about the fitness of sequences within the phylogeny. Fitness was inferred using fitness diffusion constant D = 0.5, distance scale = 2.0, and sampling fraction = 0.1. We annotated each branch with the mean fitness change from the parent to the child node. To identify branches having large fitness enhancements or diminishments, we ranked all branches by their fitness change and selected those among the top 3 or bottom 3. Our conclusions also hold true when analysis is performed using the top and bottom 1, 5, or 10 branches. We performed ancestral sequence reconstruction for each clonal lineage using maximum-likelihood assuming equal rates for all mutations. We then identified mutations that occurred on each branch by comparing the reconstructed parent and child sequences. We assigned these mutations to regions

(CDRs and FWRs) based on the region boundaries identified using IgBlast (3). To compute the enrichment of non-synonymous mutations in a region in comparison with synonymous mtuations (dN/dS), we calculated the fraction of non-synonymous mutations falling in a region, and then divided this fraction by the corresponding fraction calculated using synonymous mutations. We calculated the error of this measurement by bootstrap resampling of branches (100 replicates).

Figure S1

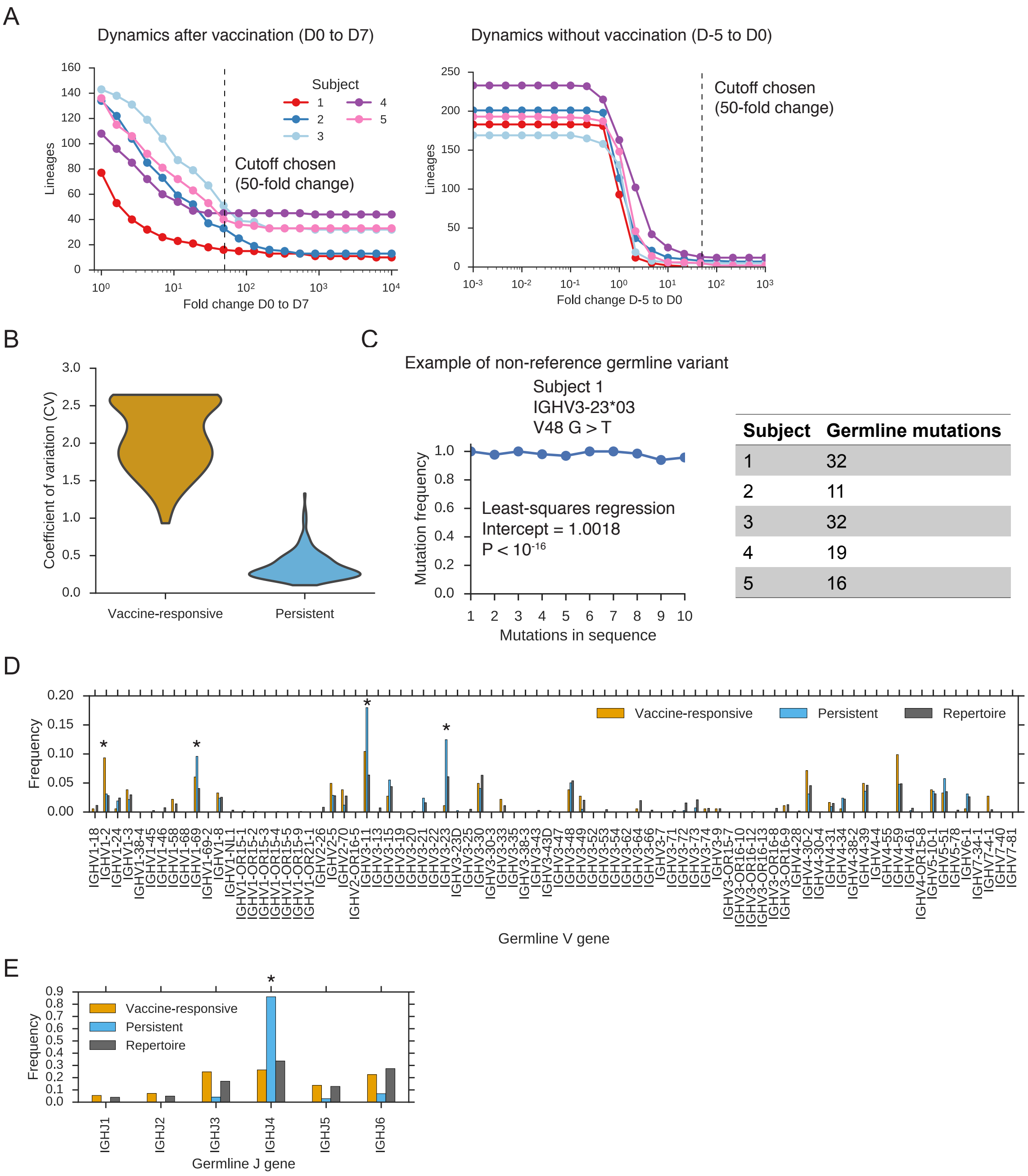

Figure S1. Dynamics of antibody repertoires and personalized annotation of germline variants.

(A) Effect of cutoff for identifying vaccine-responsive lineages. Plots show the number of lineages having a significant change in abundance as a function of the fold-change (FC) cutoff used to determine significance. Right panel, comparison of D-5 to D0 (no vaccination). Left panel, comparison of D0 to D7 (after vaccination). Dashed line indicates the cutoff of >50-fold change chosen for this work because at this value few lineages (27 within all five subjects together) are identified as having a significant change in abundance in the absence of vaccination (D-5 to D0). The changing abundance of these lineages may be due to environmental exposure to antigens, and in fact most of these lineages had undefined fold-change on the interval D-5 to D0 because they were not detected at D-5. The identity of vaccine-responsive lineages is largely

insensitive to the choice of fold-change cutoff across a broad range (10-fold to 10,000-fold increase) because most vaccine-responsive lineages are not observed at D0 and therefore have undefined fold-change on the interval D0 to D7.

(B) Dynamical variation in the fractional abundance of vaccine-responsive and persistent lineages. Plot shows the distribution of the coefficient of variation of fractional abundance for individual lineages across the observation period.

(C) Personalized annotation of germline variants for study subjects using the method of Gadala-Maria and colleagues (5). Left panel shows an example of an identified non-reference germline variant. Identification is based on the presence of a y-intercept value that is significantly larger than zero. Right panel shows the number of non-reference germline variants detected for each subject.

(D and E) Usage of germline *V* gene segments (D) or *J* gene segments (E) in vaccine-responsive or persistent lineages or the entire repertoire. Segments which are significantly overrepresented in either vaccine-responsive or persistent lineages are marked with asterisks ($P < 0.05$; Fisher's exact test, two-sided).

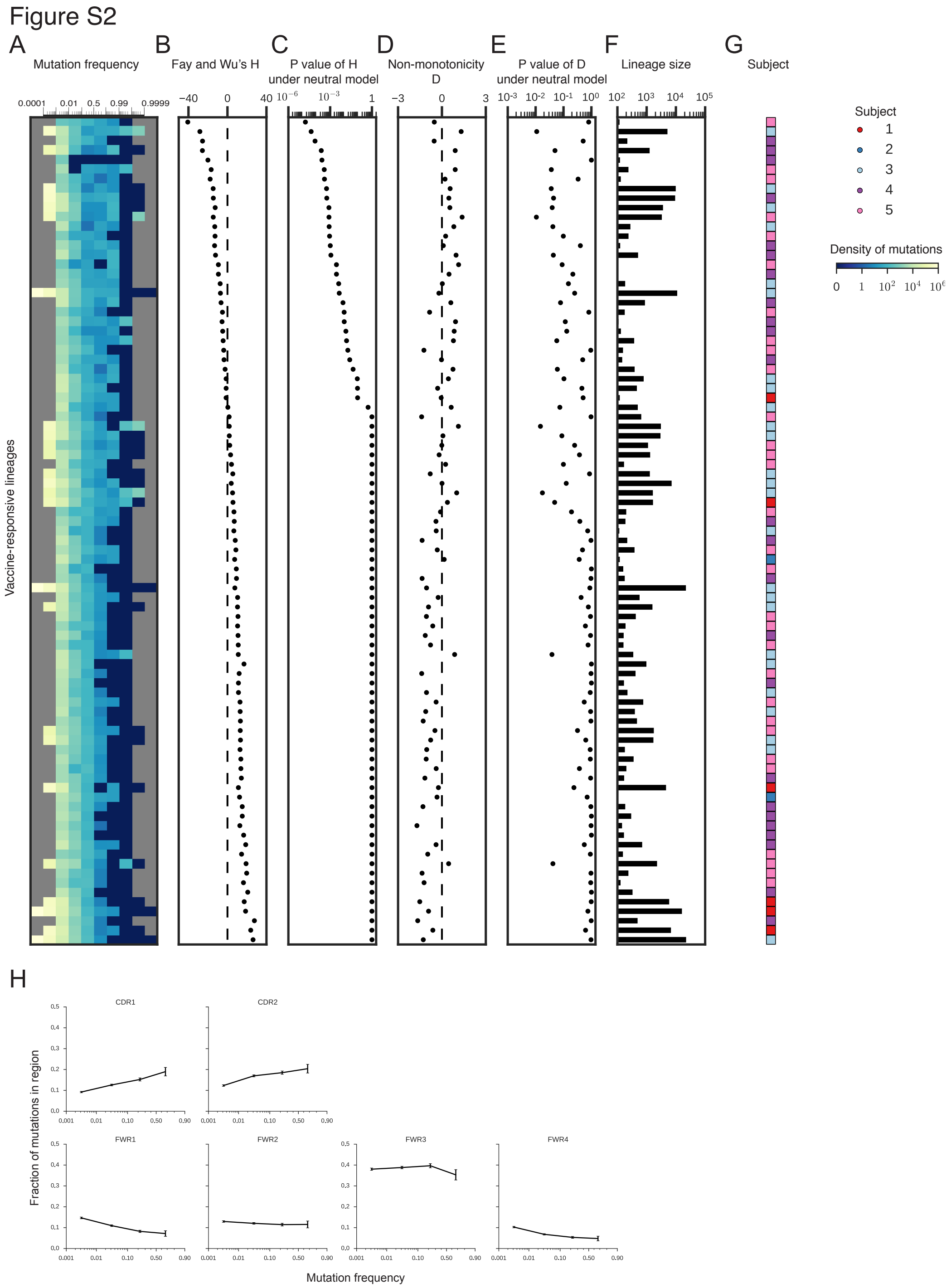

Figure S2. Genetic signatures of selection in individual vaccine-responsive B cell lineages.
(A) Site frequency spectrums (SFSs) of individual clonal vaccine-responsive B cell lineages. The density of mutations in each frequency bin is indicated by color.
(B) Fay and Wu's H statistic of each lineage.
(C) Significance of Fay and Wu's H statistic in comparison with a null model of neutral drift.
(D) Non-monotonicity D of the SFS of each lineage.
(E) Significance of the non-monotonicity D in comparison with a null model of neutral drift.
In (C) and (E), significance values were calculated by creating a ensemble of lineages via simulation of the Kingman coalescent model (neutral drift-like evolution with constant population

size) with each lineage having a population size matching that of the focal lineage, calculating the desired test statistic on each simulated lineage, fitting the Johnson's U distribution to the simulated distribution of test statistics, then calculating the P value of the observed value of the test statistic.
(F) Number of sequences in each lineage observed at D7 using long amplicon sequencing (paired-end 300 bp sequencing of 480 bp amplicons).
(G) Subject of origin of each clonal B cell lineage.
(H) Distribution of mutations across sequence regions for mutations of different frequencies found in vaccine-responsive lineages. All mutations were placed into bins based on their frequency, then within each bin the fraction of mutations falling in each region was calculated. Error bars show exact binomial 95% confidence intervals.

Figure S3

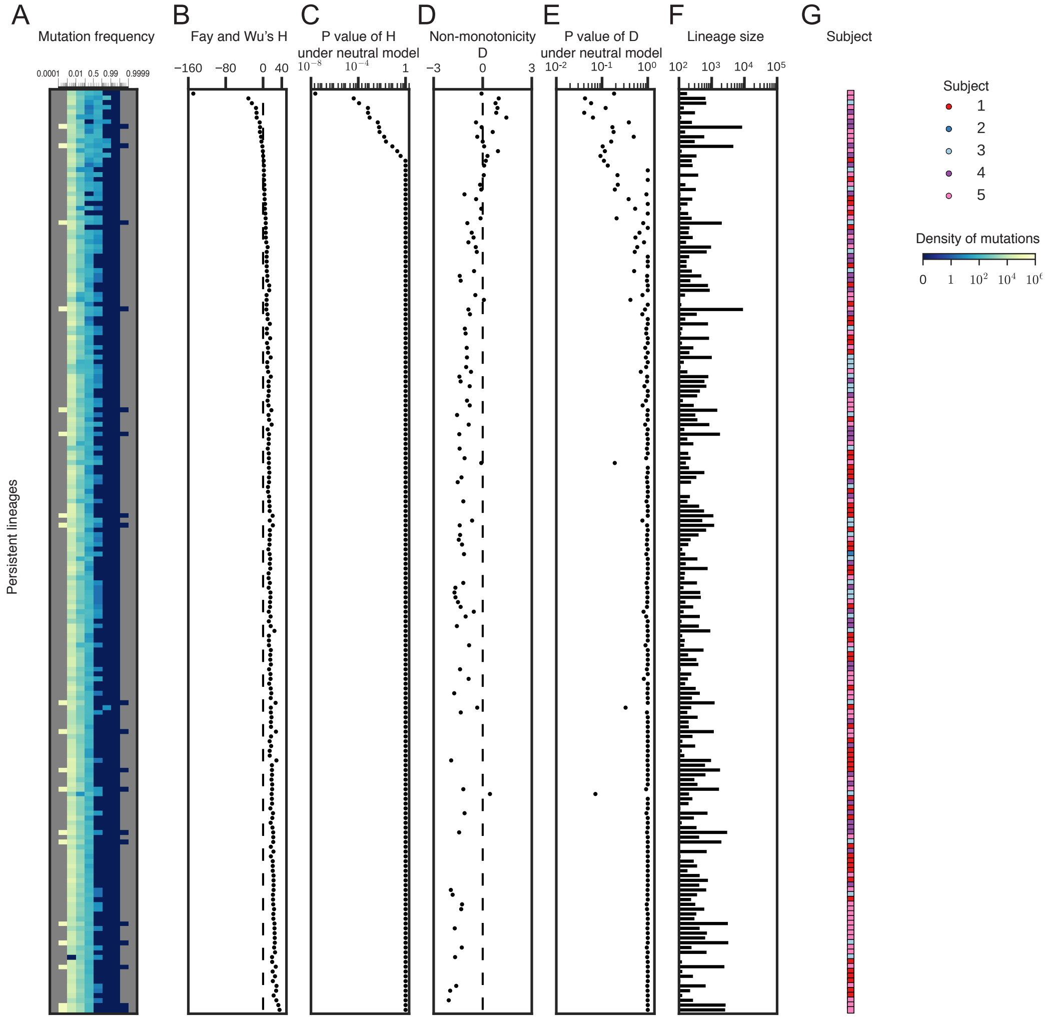

Figure S3. Genetic signatures of neutral evolution in individual persistent B cell lineages.
(A) Site frequency spectrums (SFSs) of individual clonal persistent B cell lineages. The density of mutations in each frequency bin is indicated by color.
(B) Fay and Wu's H statistic of each lineage.
(C) Significance of Fay and Wu's H statistic in comparison with a null model of neutral drift.
(D) Non-monotonicity D of the SFS of each lineage.
(E) Significance of the non-monotonicity D in comparison with a null model of neutral drift.
In (C) and (E), significance values were calculated as described in Figure S2.
(F) Number of sequences in each lineage observed at D7 using long amplicon sequencing (paired-end 300 bp sequencing of 480 bp amplicons).
(G) Subject of origin of each clonal B cell lineage.

Figure S4

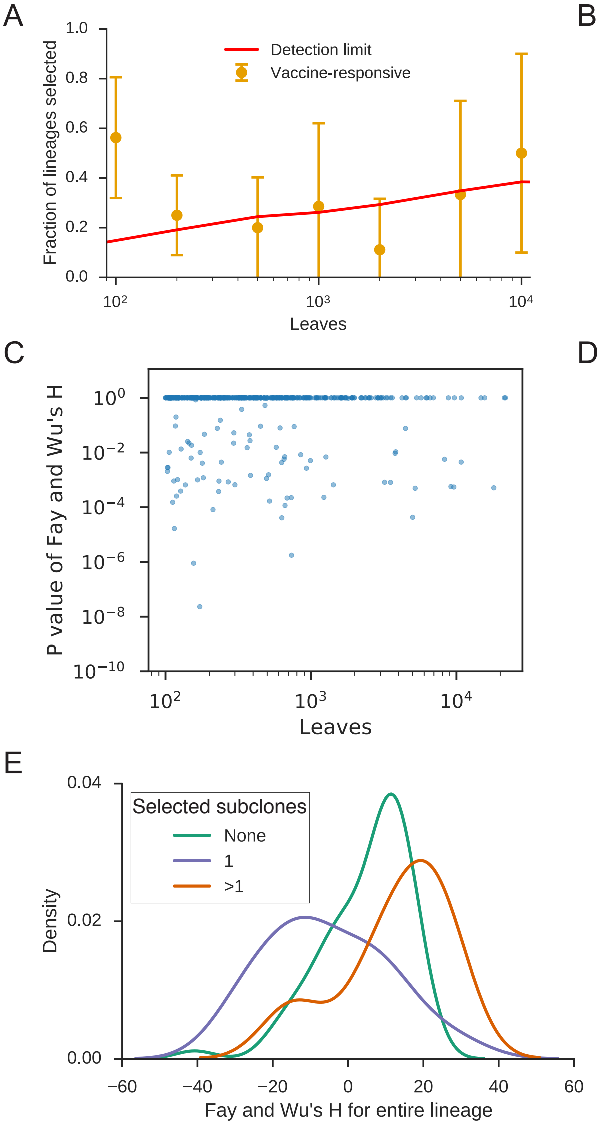

Figure S4. Limits of detection on selection.
(A and B) Rate of detecting selection among vaccine-responsive (A) and persistent (B) lineages of varying size. Detection limit imposed by population size is shown for comparison, assuming a false discovery rate (FDR) of 0.05. Error bars show exact binomial 95% confidence intervals.
(C) Relationship between lineage size and signatures of selection (by comparison with neutral model without population expansion). Each dot is a lineage.
(D) Relationship between lineage size and total genetic diversity (measured as total branch length in phylogeny). Each dot is a lineage and is colored to indicate whether it is vaccine-responsive or persistent (as in A and B).
(E) Distributions of Fay and Wu's H statistic for vaccine-responsive lineages in which one, multiple, or no subclones have evidence for positive selection (FDR = 1%). Lineages in which multiple subclones were selected display a rightward shift in the distribution of H, reflecting the hard upper bound on the frequency of mutations that are private to each subclone and causing this test for selection to fail when applied to the entire lineage.

Figure S5

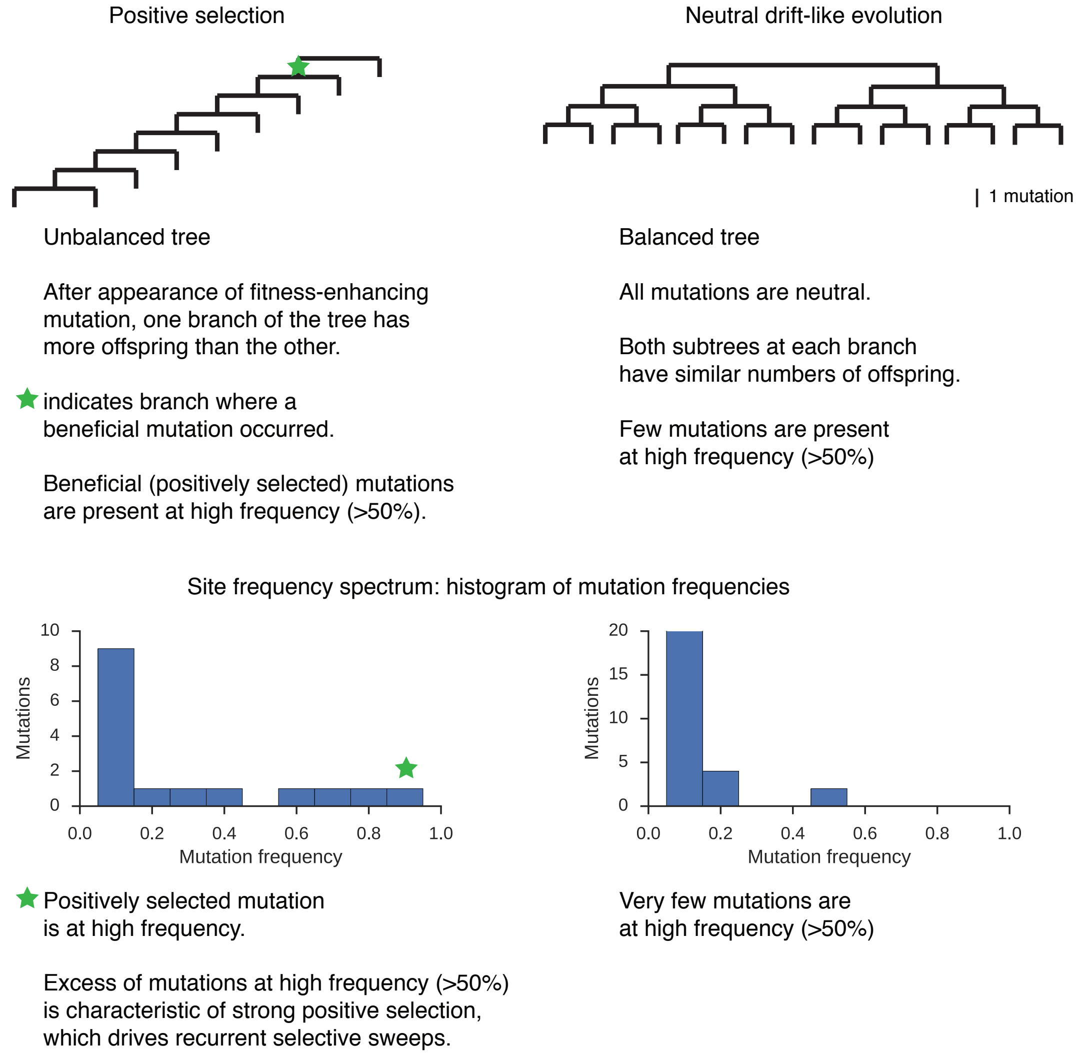

Figure S5. Explanation of relationships between positive selection, neutrality, phylogenetic tree shape, and the site-frequency spectrum (SFS).

Schematic illustrating how the shape of phylogenetic trees (top panels) and the site frequency spectrum (SFS) (bottom panels) differ between populations evolving under positive selection (left panels) and neutral drift (right panels). In populations undergoing continuous adaptation driven by positive selection, repeated appearance of beneficial mutations leads to an unbalanced tree shape. Beneficial mutations are present at high frequency. In contrast, in populations evolving neutrally, all mutations are neutral and do not confer any fitness benefit. Thus, similar numbers of progeny are present on each branch, leading to a balanced tree shape. The SFS is a histogram of the mutation frequencies in the population. During continuous adaptation driven by positive selection, beneficial mutations become present at high frequency, unlike in populations evolving neutrally. Thus, an excess of mutations at high frequency is a characteristic signature of strong positive selection driving recurrent selective sweeps.

Table S1. Vaccine-responsive and persistent lineages found in each subject.

Subject	Vaccine-responsive lineages	Persistent lineages	Total lineages
1	16	111	55,545
2	32	97	30,823
3	49	44	41,633
4	45	76	18,104
5	40	89	23,263

Table S2. PCR primers used for library preparation.
Short amplicon primers were used to prepare libraries for paired-end 100 bp sequencing from samples from all time points. Long amplicon primers were used to prepare libraries for paired-end 300 bp sequencing from samples from D7. RT, reverse transcription; SS, second-strand synthesis.

Amplicon type	Step	Name	Sequence (5’–3’)
Short	RT	G	TGACTGGAGTTCAGACGTGTGCTCTTCCGATCTNNNNNNNNAAGACCGATGGGCCCTTG
		A	TGACTGGAGTTCAGACGTGTGCTCTTCCGATCTNNNNNNNNGAAGACCTTGGGGCTGGT
		M	TGACTGGAGTTCAGACGTGTGCTCTTCCGATCTNNNNNNNNGGGAATTCTCACAGGAGACG
		D	TGACTGGAGTTCAGACGTGTGCTCTTCCGATCTNNNNNNNNGGGTGTCTGCACCCTGATA
		E_1	TGACTGGAGTTCAGACGTGTGCTCTTCCGATCTNNNNNNNNGAAGACGGATGGGCTCTGT
		E_2	TGACTGGAGTTCAGACGTGTGCTCTTCCGATCTNNNNNNNNTTGCAGCAGCGGGTCAAGGG
	SS	V1	ACACTCTTTCCCTACACGACGCTCTTCCGATCTNNNNNNNNAGCCTACATGGAGCTGAGC
		V2	ACACTCTTTCCCTACACGACGCTCTTCCGATCTNNNNNNNNAGGTGGTCCTTACAATGACCAAC
		V3_1	ACACTCTTTCCCTACACGACGCTCTTCCGATCTNNNNNNNNTCTGCAAATGAACAGCCTGA
		V3_2	ACACTCTTTCCCTACACGACGCTCTTCCGATCTNNNNNNNNTGTTCAAATGAGCAGTCTGAGAG
		V3_3	ACACTCTTTCCCTACACGACGCTCTTCCGATCTNNNNNNNNTCTGCAAATGGGCAGCCTGA
		V4/6	ACACTCTTTCCCTACACGACGCTCTTCCGATCTNNNNNNNNTTCTCCCTGAAGCTGAACTCTG
		V5	ACACTCTTTCCCTACACGACGCTCTTCCGATCTNNNNNNNNGCCTACCTGCAGTGGAGCAG
		V6	ACACTCTTTCCCTACACGACGCTCTTCCGATCTNNNNNNNNTTCTCCCTGCAGCTGAACTCTG
		V7_1	ACACTCTTTCCCTACACGACGCTCTTCCGATCTNNNNNNNNGCATATCTGCAGATCAGCAGC
		V7_2	ACACTCTTTCCCTACACGACGCTCTTCCGATCTNNNNNNNNCAGATCAGCAGCCTAAAGGC
Long	RT	IgA_08N	TGACTGGAGTTCAGACGTGTGCTCTTCCGATCTNNNNNNNNGGGGAAGAAGCCCTGGAC
		IgA_12N	TGACTGGAGTTCAGACGTGTGCTCTTCCGATCTNNNNNNNNNNNNGGGGAAGAAGCCCTGGAC
		IgG_08N	TGACTGGAGTTCAGACGTGTGCTCTTCCGATCTNNNNNNNNGGGAAGTAGTCCTTGACCA
		IgG_12N	TGACTGGAGTTCAGACGTGTGCTCTTCCGATCTNNNNNNNNNNNNGGGAAGTAGTCCTTGACCA
		IgM_long_8N	TGACTGGAGTTCAGACGTGTGCTCTTCCGATCTNNNNNNNNGAAGGAAGTCCTGTGCGAG
		IgM_long_12N	TGACTGGAGTTCAGACGTGTGCTCTTCCGATCTNNNNNNNNNNNNGAAGGAAGTCCTGTGCGAG
		IgE_long_8N	TGACTGGAGTTCAGACGTGTGCTCTTCCGATCTNNNNNNNNAAGTAGCCCGTGGCCAGG
		IgE_long_12N	TGACTGGAGTTCAGACGTGTGCTCTTCCGATCTNNNNNNNNNNNNAAGTAGCCCGTGGCCAGG
		IgD_long_8N	TGACTGGAGTTCAGACGTGTGCTCTTCCGATCTNNNNNNNNTGGGTGGTACCCAGTTATCAA
		IgD_long_12N	TGACTGGAGTTCAGACGTGTGCTCTTCCGATCTNNNNNNNNNNNNTGGGTGGTACCCAGTTATCAA
	SS	V1_1_70	ACACTCTTTCCCTACACGACGCTCTTCCGATCTNNNNNNNNSCAGCTGGTGCAGTCTGG
		V1/3/5_70	ACACTCTTTCCCTACACGACGCTCTTCCGATCTNNNNNNNNGTGCAGCTGGTGGAGTCTG
		V2_70	ACACTCTTTCCCTACACGACGCTCTTCCGATCTNNNNNNNNGTGCAGCTGGTGGAGTCTG
		V4_1_70	ACACTCTTTCCCTACACGACGCTCTTCCGATCTNNNNNNNNTGCAGCTGCAGGAGTCG
		V4_2_70	ACACTCTTTCCCTACACGACGCTCTTCCGATCTNNNNNNNNGTGCAGCTACAGCAGTGG
		V6_70	ACACTCTTTCCCTACACGACGCTCTTCCGATCTNNNNNNNNGTACAGCTGCAGCAGTCA

References

1. Vollmers C, Sit RV, Weinstein JA, Dekker CL, Quake SR (2013) Genetic measurement of memory B-cell recall using antibody repertoire sequencing. *PNAS* 110(33):13463–13468.
2. Horns F, et al. (2016) Lineage tracing of human B cells reveals the in vivo landscape of human antibody class switching. *eLife Sciences* 5:e16578.
3. Ye J, Ma N, Madden TL, Ostell JM (2013) IgBLAST: an immunoglobulin variable domain sequence analysis tool. *Nucleic Acids Res* 41(Web Server issue):W34–W40.
4. Gupta NT, et al. (2017) Hierarchical Clustering Can Identify B Cell Clones with High Confidence in Ig Repertoire Sequencing Data. *J Immunol* 198(6):2489–2499.
5. Gadala-Maria D, Yaari G, Uduman M, Kleinstein SH (2015) Automated analysis of high-throughput B-cell sequencing data reveals a high frequency of novel immunoglobulin V gene segment alleles. *PNAS* 112(8):E862–E870.
6. Neher RA, Kessinger TA, Shraiman BI (2013) Coalescence and genetic diversity in sexual populations under selection. *PNAS* 110(39):15836–15841.
7. Fay JC, Wu C-I (2000) Hitchhiking Under Positive Darwinian Selection. *Genetics* 155(3):1405–1413.
8. Edgar RC (2004) MUSCLE: multiple sequence alignment with high accuracy and high throughput. *Nucl Acids Res* 32(5):1792–1797.
9. Teng G, Papavasiliou FN (2007) Immunoglobulin somatic hypermutation. *Annu Rev Genet* 41:107–120.
10. McCoy CO, et al. (2015) Quantifying evolutionary constraints on B-cell affinity maturation. *Phil Trans R Soc B* 370(1676):20140244.
11. Price MN, Dehal PS, Arkin AP (2010) FastTree 2 – Approximately Maximum-Likelihood Trees for Large Alignments. *PLoS ONE* 5(3):e9490.
12. Neher RA, Russell CA, Shraiman BI (2014) Predicting evolution from the shape of genealogical trees. *eLife Sciences* 3:e03568.
